# The Effects of Social and Demographic Factors on High-Volume Hospital and Surgeon Care in Shoulder Arthroplasty

**DOI:** 10.5435/JAAOSGlobal-D-22-00107

**Published:** 2022-08-12

**Authors:** Edward J. Testa, Peter G. Brodeur, Kang Woo Kim, Jacob M. Modest, Cameron W. Johnson, Aristides I. Cruz, Joseph A. Gil

**Affiliations:** From the Department of Orthopaedic Surgery, Brown University, Warren Alpert School of Medicine, Providence, RI.

## Abstract

This study seeks to evaluate (1) the relationship between hospital and surgeon volumes of shoulder arthroplasty and complication rates and (2) patient demographics/socioeconomic factors that may affect access to high-volume shoulder arthroplasty care.

**Methods:**

Adults older than 40 years who underwent shoulder arthroplasty between 2011 and 2015 were identified in the New York Statewide Planning and Research Cooperative System database using International Classification of Disease 9/10 and Current Procedural Terminology codes. Medical/surgical complications were compared across surgeon and facility volumes. The effects of demographic factors were analyzed to determine the relationship between such factors and surgeon/facility volume in shoulder arthroplasty.

**Results:**

Seven thousand seven hundred eighty-five patients were included. Older, Hispanic/African American, socially deprived, nonprivately insured patients were more likely to be treated by low-volume facilities. Low-volume facilities had higher rates of readmission, urinary tract infection, renal failure, pneumonia, and cellulitis than high-volume facilities. Low-volume surgeons had patients with longer hospital lengths of stay.

**Discussion:**

Important differences in patient socioeconomic factors exist in access to high-volume surgical care in shoulder arthroplasty, with older, minority, and underinsured patients markedly more likely to receive care by low-volume surgeons and facilities. This may highlight an area of potential focus to improve access to high-volume care.

Healthcare costs in the United States continue to rise with a 2017 national health expenditure report placing annual costs at $3.5 trillion, which equals 17.9% of the nation's gross domestic product.^1,2^ Owing to their high volume and notable expenditure, large joint arthroplasty procedures have become a key focus of cost analyses.^3-5^ Cost control strategies have been extensively investigated within hip and knee arthroplasty, with one facet of such research focused on establishing minimum provider and facility volume standards to ensure these goals.^1,6^ Over the past decade, a similar interest has been growing for total shoulder arthroplasty (TSA), a reconstructive procedure which has experienced a sharp increase in surgical volume over recent years.^5,7^

Although the relationship between surgeon and hospital volumes and patient outcomes has been thoroughly investigated in lower extremity arthroplasty and trauma procedures, this relationship has yet to be fully elucidated in TSA procedures.^8,9^ Previous studies have associated higher surgeon and hospital TSA volumes with reduced length of stay and complication rates;^10^ however, few have evaluated the effect of patient demographics and the disparities that exist in the access to shoulder arthroplasty.^11,12^ Key patient demographic factors, including race, ethnicity, and insurance status, have been variably implicated as factors affecting access to quality care and patient outcomes in the field of orthopaedic surgery.^13,14^ In addition, the current body of literature lacks specific details on the complications that are associated with surgeon and hospital volumes.^18^

Because of the notable increases observed in the volume of both primary anatomic and reverse TSAs nationally, projections currently estimate volume increases of up to 235% by 2025.^1^ Therefore, evaluating the relationship between patient demographic factors, access to care, postoperative outcomes, and surgeon volume is important.^7,12^ The purpose of this study was twofold: We sought to (1) evaluate the relationship between complications and postoperative outcomes and surgeon and hospital volumes after total shoulder arthroplasty and (2) assess patient factors associated with access to surgical care for shoulder arthroplasty. Our hypotheses were that surgeon and hospital volumes are inversely correlated with patient complications, and minority patients with higher social deprivation indexes and patients with nonprivate insurance types would be more likely to be treated at low-volume hospitals by low-volume surgeons.

## METHODS

Patients aged 40 years and older were identified in the New York Statewide Planning and Research Cooperative System (SPARCS) database from 2011 to 2015. This is an all-payer database collecting outpatient (emergency department, ambulatory surgery, and hospital-based clinic visits) and all inpatient claims in New York state, which includes all International Classification of Diseases (ICD) diagnosis codes and ICD/Current Procedural Terminology procedure codes associated with all visits. Inpatient surgery claims were first identified using the ICD-9 Clinical Modification glenohumeral arthritis diagnosis codes (715.11, 715.21, 715.31, 715.91, 716.51, and 716.61). Claims were then filtered by ICD-9 Clinical Modification procedure codes to isolate patients who went on to receive a shoulder arthroplasty procedure (81.80, 81.81, and 81.88). Only a patient's first operation was eligible for follow-up. Nonresidents of New York were not included in our analysis. Given that ICD-9 coding was discontinued after the third quarter of 2015, only the first 3 quarters of 2015 were used because these statistics are still likely to be indicative of the low-volume to high-volume comparison.

Each surgeon had their own unique identifier, which was used to calculate the total number of procedures per surgeon annually. Based on total annual volume, surgeons were assigned to the lowest 20% of the volume, middle 60% of the volume, or highest 20% of the volume. The boundaries for lowest and highest 20% deviated slightly by year but were selected to minimize the difference from the 20% volume mark. This technique has previously been used in volume analysis of peritrochanteric hip fractures.^9^

Patients were followed up for 1 year postoperatively in both the inpatient and outpatient settings. The 1-, 3-, and 12-month risks of interest were readmission, urinary tract infection, acute renal failure, cardiorespiratory arrest, pneumonia, acute stroke, surgical site infection, deep vein thrombosis, acute respiratory failure, pulmonary embolism, cellulitis, wound complications, mortality, and revision surgery (see Supplemental Table 1, http://links.lww.com/JG9/A230, for codes used). An additional analysis was conducted to determine the effect of facility or surgeon volume on hospital length of stay. Statewide Planning and Research Cooperative System claims dates are listed as the first day of the month when the service occurred because of the SPARCS deidentification policy. Therefore, if a complication occurred within the same month as the primary procedure, the time to complication was defined as 0.5 months.^9^

Patient demographics were compared across facility volume and surgeon volume using chi-square analysis. Student *t*-tests were used for comparing sample means, and Mann-Whitney *U* tests were used when appropriate when continuous data were found to be not normally distributed. The rates of complications were compared separately across facility volume and surgeon volume.

Multivariable Cox proportional hazards regression was used for the analysis of risk likelihood across the volume groups. Each complication was modeled separately while controlling for patient age, sex, race, ethnicity, Charlson Comorbidity Index, primary insurance type, and social deprivation index (SDI). “Other race” excludes White, Asian, and African American but does include multiracial. The regression models assess the risk difference across surgeon and facility groups simultaneously by controlling for both in the same model. A linear regression model using the same independent variables was used to determine the effect of surgeon or facility volume on length of stay.

The Charlson Comorbidity Index was calculated using the described method of Deyo et al.^16^ The Charlson Comorbidity Index was dichotomized to a score of 0 versus a score of ≥1. The social deprivation index as described by Butler et al^17^ was linked to each patient based on ZIP codes. The social deprivation index provides a measure of the social determinants of health that are not commonly captured by healthcare databases by converting certain categories to an index ranging from 1 to 100. These categories include percent living in poverty, percent living in rented housing unit, percent with less than 12 years of education, percent single-parent household, percent living in overcrowded housing unit, percent of households without a car, and percent nonemployed adults younger than 65 years. Thus, higher SDI scores equate to increased social deprivation. Social deprivation index data in this study were based on 2015 statistics. A *P*-value of <0.05 was considered significant across all statistical analyses. All analyses were conducted using SAS 9.4 (SAS Inc).

## RESULTS

Seven thousand seven hundred eighty-five patients were identified, 4,762 of which were treated by a high or low-volume facility or surgeon. Annual facility volume ranged from 1 to 348 procedures (mean: 15, median: 6). Annual surgeon volume ranged from 1 to 109 procedures (mean: 6, median: 3). The number of procedures per year in New York was 1,422 in 2011 and 1,638 in 2014 (1,616 through 3 quarters of 2015). Low-volume facilities accounted for 1,735 procedures, and high-volume facilities accounted for 1,714 procedures. Low-volume surgeons accounted for 1,666 procedures, and high-volume surgeons accounted for 1,642 procedures (Tables 1 and 2).

**Table 1:** Patient Demographics and Characteristics by Facility Volume

	Low Volume n = 1,735	High Volume n = 1,714	*P*
Age, mean (SD)	69.1 (70, 10.1)	68.3 (69, 9.5)	**0.022**
Sex, n (%)			
Female	955 (55)	935 (54.6)	0.7714
Male	780 (45)	779 (45.5)	—
Ethnicity, n (%)			
Non-Hispanic	1,576 (90.8)	1,672 (97.6)	**<0.0001**
Hispanic	159 (9.2)	42 (2.5)	—
Race, n (%)			
White	1,453 (83.8)	1,494 (87.2)	**0.0044**
Asian	8 (0.5)	2 (0.1)	**0.06**
African American	148 (8.5)	66 (3.9)	**<0.0001**
Other	126 (7.3)	152 (8.9)	0.0832
Primary insurance, n (%)			
Private	524 (30.2)	743 (43.4)	**<0.0001**
Federal	1,089 (62.8)	899 (52.5)	**<0.0001**
Self-pay	25 (1.4)	1 (0.1)	**<0.0001**
Charlson score, n (%)			
0	927 (53.4)	1,022 (59.6)	**0.0002**
≥1	808 (46.6)	692 (40.4)	—
SDI, median (mean, SD)	40 (44.6, 29.7)	32 (37.5, 28.7)	**<0.0001**

SDI = social deprivation index. Bold denotes statistical significance at P<0.05.

**Table 2:** Patient Demographics and Characteristics by Surgeon Volume

	Low Volume n = 1,666	High Volume n = 1,642	*P*
Age, mean (SD)	68.5 (69, 10.1)	68.5 (69, 9.4)	0.8453
Sex, n (%)			
Female	915 (54.9)	921 (56.1)	0.4991
Male	751 (45.1)	721 (43.9)	—
Ethnicity, n (%)			
Non-Hispanic	1,521 (91.3)	1,614 (98.3)	**<0.0001**
Hispanic	145 (8.7)	28 (1.7)	—
Race, n (%)			
White	1,339 (80.4)	1,462 (89)	**<0.0001**
Asian	13 (0.8)	2 (0.1)	**0.0048**
African American	133 (8)	51 (3.1)	**<0.0001**
Other	181 (10.9)	127 (7.7)	**0.002**
Primary insurance, n (%)			
Private	539 (32.4)	648 (39.5)	**<0.0001**
Federal	990 (59.4)	919 (56)	**0.0443**
Self-pay	28 (1.7)	3 (0.2)	**<0.0001**
Charlson score, n (%)			
0	905 (54.3)	933 (56.8)	0.1481
≥1	761 (45.7)	709 (43.2)	—
SDI, median (mean, SD)	41 (45.8, 30.1)	31 (36.7, 28.5)	**<0.0001**

SDI = social deprivation index. Bold denotes statistical significance at P<0.05.

Older patients were more commonly treated at low-volume facilities compared with high-volume ones. Both low-volume facilities and surgeons had patients with higher SDI scores relative to high-volume facilities and surgeons (Tables 1 and 2). Patients of Hispanic ethnicity, Asian race, and African American race; those with Federal insurance and self-pay; and those having Charlson Comorbidity Index ≥1 were more likely to be cared for at low-volume facilities (Table 1). Low-volume surgeons were more likely to care for patients of Hispanic ethnicity, Asian race, African American race, and other race; those with Federal insurance; and those who are self-pay (Table 2).

Compared with high-volume facilities, low-volume facilities were noted to have higher 1-, 3-, and 12-month rates of readmission and acute renal failure, higher 3- and 12-month rates of urinary tract infection, higher 12-month rates of pneumonia and cellulitis, and lower 3-month rate of acute stroke (Supplemental Table 2, http://links.lww.com/JG9/A231). Compared with high-volume surgeons, patients of low-volume surgeons had lower 3-month rates of acute renal failure (Supplemental Table 3, http://links.lww.com/JG9/A232). Patients of low-volume surgeons had longer hospital lengths of stay than those of higher volume (*P* = 0.0076).

**Figure 1:**
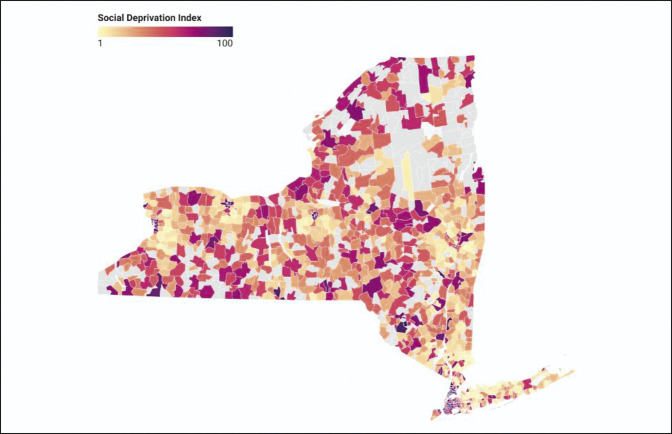
Diagram showing the social deprivation index by New York ZIP code. Gray ZIP codes had no shoulder arthroplasty cases during the study period.

**Figure 2:**
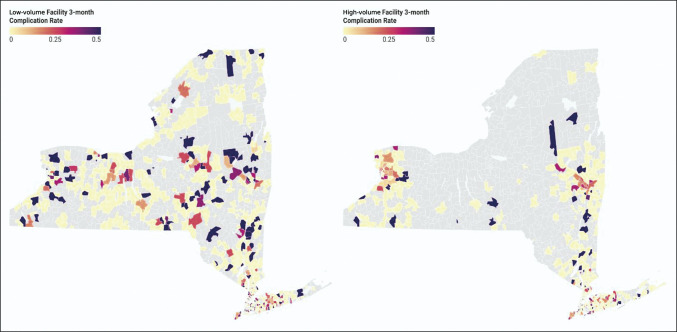
Diagram showing 3-month complication rates by facility and surgeon volumes by ZIP codes. Gray ZIP codes had no complications during the study period.

**Figure 3:**
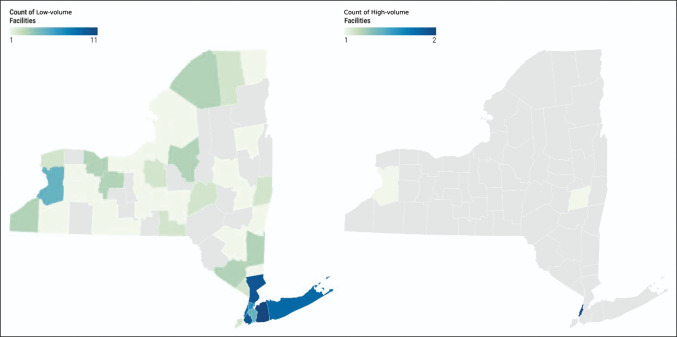
Diagram showing density of high and low-volume centers in New York by county. Gray county codes had either no facilities or middle-volume facilities.

Patients who were treated at low-volume facilities and proceeded to have complications were more concentrated in areas with higher SDI scores, whereas patients treated at high-volume facilities who experienced complications had lower SDI scores (Figures 1 and 2). Patients treated at low-volume facilities and proceeded to have complications were also more widely distributed across New York state (Figure 2). There was a disproportionate amount of low-volume facilities in areas with the highest SDI scores: Western New York, Northern New York, and Western Long Island (Figure 3).

## DISCUSSION

The relationship between hospital and surgeon volumes and surgical outcomes has been extensively studied throughout the field of orthopaedic surgery, including shoulder arthroplasty.^8,13,18–21^ Given the current body of literature which suggests that, in general, higher volume is associated with superior surgical outcomes, it is important to identify patient factors associated with undergoing surgery with low-volume surgeons or providers. Moreover, there is little literature evaluating the relationship between hospital and surgeon volumes and patient demographic factors in shoulder arthroplasty. Our analysis demonstrates that there are differences in complication rates and types of complications between high and low-volume facilities and surgeons in shoulder arthroplasty. In addition, there are also differences among various patient demographic groups regarding receiving high-volume care, which is in accordance with our hypothesis. This, in turn, may lead to differences in patient outcomes among such patient groups and may highlight an area of potential focus to improve access to high-quality care.

A comparison of postoperative medical outcomes after TSA demonstrated that low-volume facilities cared for patients with higher rates of acute renal failure, urinary tract infection, and pneumonia, but lower rates of acute stroke. Regarding surgeon volume, patients of low-volume surgeons had increased hospital length of stay, but with lower risk of acute renal failure. The increased rates of certain medical complications, such as acute renal failure, urinary tract infection, and pneumonia, noted in patients cared for at low-volume facilities align with the results of a recent large database study concluding that the most common causes of readmission after shoulder arthroplasty were pulmonary problems (21%), electrolyte imbalance (28%), and urinary tract infections (10%).^22^ Furthermore, the increased hospital length of stay noted in our study parallels a 2014 study that similarly showed an inverse relationship between length of stay and surgeon volume.^25^

The results of this study highlight the variation in shoulder arthroplasty surgeon and facility utilization among various patient races. Our findings demonstrate that Hispanic ethnicity, Asian race, and African American race were more likely to receive care at low-volume facilities and by low-volume surgeons. Tompson et al^24^ previously reported on a large sample of patients undergoing shoulder arthroplasty and noted substantial disparities between Black and non-Black patients at the national level, with markedly higher utilization in non-Black patients, despite similar rates of osteoarthritis within such demographics. The relationship between patient race and TSA procedure volume and utilization have been similarly explored in other research, with several authors stating that non-White and Hispanic patients were more likely to undergo fewer TSA procedures or receive them at lower volume hospitals.^27^ Furthermore, in the New York metropolitan area, Black and Hispanic patients have been reported to undergo hip arthroplasty less frequently with high-volume hospitals and surgeons.^26^ Ultimately, these racial and ethnic differences observed in this study among high and low-volume surgeons and facilities afford a potential opportunity in improving equity in access to high-quality surgical care.

Our findings also suggest that there may be an increased complication burden on the patients who receive care with lower volume hospitals or providers; therefore, it is important to identify these vulnerable populations. Garcia et al^27^ found that Black patients had a 45% higher likelihood of a 3-month emergency department visit after primary shoulder arthroplasties; Jain et al^28^ reported that patients with comorbidities such as hypertension and diabetes had an increased risk of postoperative complications. Because our study found that these patient populations were also more likely to receive care at low-volume centers, there is the concern that these patients will be the most disadvantaged by the differences in outcomes. Our study also found that patients without private insurance received treatment more often at low-volume facilities and from low-volume surgeons, which is another concerning finding considering that surgeon reimbursement for Medicare payments after shoulder arthroplasty has decreased substantially over the past decade.^29^ It is also important to acknowledge the possible influence of the geographic location of hospitals alongside any financial incentives for high-volume providers to attract patients with insurances which offer higher reimbursement rates, most of whom may be White.^11^

Overall, both low-volume hospitals and surgeons cared for an increased number of patients classified as Hispanic, non-White race, and underinsured patients, and the related reduced access to care and increased risk of postoperative complications align with the literature.^30-32^ Thus, there is a need to explore methods of increasing access and reducing the differences between high and low-volume care. A review of the 2012 Medicare Provider Utilization and Payment Data Public Use File showed that in 2012, there were 21 shoulder arthroplasty surgeons in New York state who submitted more than 11 Medicare claims for TSA, and the number of surgeons conducting more than 10 shoulder arthroplasties annually has increased since 1998. This boost may be attributed to the fact that by 2017, New York City had the greatest number of annual American Shoulder and Elbow Surgeons fellowship positions within the metropolitan area with total five positions. In addition, metropolitan areas with American Shoulder and Elbow Surgeons fellowship positions had both markedly more high-volume TSA surgeons and markedly greater number of Medicare claims for TSA.^32^ Thus, there may be a benefit in establishing robust educational programs for surgeons conducting these procedures because this will minimize the potential adverse effects of low surgeon volume for both patients and the economy of the healthcare system. It is also important to recognize the greater effect of hospital volumes compared with that of surgeon volumes on patient outcomes; hospital volume as a metric can better encompass protocolized pathways, ancillary staff familiarity, and other related logistics that affect patient care.^33^ Because high-volume hospitals have a greater capacity for care, they may consequently be better equipped to proactively identify and resolve issues before they escalate and adversely influence patient outcomes; this emphasizes the value and importance of equal access to care for all patients.

There are several limitations to this study. The use of a large database inherently requires accurate coding. Because this study evaluated outcomes for the same procedure across the database, any differences in reporting should be global, and the large sample size should help minimize substantial changes to the observed outcomes. Moreover, there are several significant demographic differences between the cohort included in this study (Tables 1 and 2), although we did attempt to control for these during our statistical analysis. Our study involved patients within the confined geographic zone of the SPARCS database. Therefore, national and global trends cannot be directly assessed, possibly limiting appropriate extrapolation to other areas. However, New York is a large state composed of a highly variable population of patients, hospitals, and surgeons with a great degree of demographic variability and therefore may be generalizable to larger populations. Finally, the thresholds representing high or low volumes were determined based on a previously used statistical technique from the hip fracture literature^9^ and thus could lead to altered outcomes if these thresholds were established by another technique.

In conclusion, there is disparate access to high-volume surgical care relating to socioeconomic factors in shoulder arthroplasty, which is an important finding warranting additional investigation and resolution. The importance of case volume in shoulder arthroplasty is relevant for both facilities and providers. Although there were no overarching uniform relationships noted among medical or surgical complications as a function of hospital and surgeon volumes, low-volume facilities were associated with increased risks for complications such as urinary tract infection and acute renal failure while low-volume surgeons were associated with an increased length of stay. Our results suggest that volume shifting toward higher volume facilities and/or surgeons could improve patient outcomes and that changes at the systemic level may be necessary to enhance access to high-volume surgical care in shoulder arthroplasty for minority patients in more socially deprived locations.
